# Prognostic Value of the NAPLES Score and Serum Uric Acid in Chronic Coronary Syndrome: Evidence from Time-Dependent ROC and Time-Varying Hazard Ratio Analyses

**DOI:** 10.3390/jcm14207416

**Published:** 2025-10-20

**Authors:** Seda Elcim Yildirim, Tarik Yildirim, Tuncay Kiris, Eyüp Avci

**Affiliations:** 1 Department of Cardiology, School of Medicine, Balikesir University, Balikesir 10145, Turkey; sedaelcimdurusoy@gmail.com (S.E.Y.); kdrtarik@gmail.com (T.Y.); dreyupavci@gmail.com (E.A.); 2 Department of Cardiology, Atatürk Training and Research Hospital, Izmir Katip Çelebi University, Basin Site, Karabaglar 35360, Turkey

**Keywords:** chronic coronary syndromes, major adverse cardiovascular and cerebrovascular events (MACCE), uric acid, Naples score

## Abstract

**Background and Objectives**: The Naples Prognostic Score (NPS), a composite index indicative of nutritional and inflammatory status, has been suggested as an important prognostic marker. Uric acid, an indicator of oxidative stress and endothelial impairment, is also associated with cardiovascular risk. This study sought to examine the synergistic value of NPS and uric acid levels in forecasting long-term major adverse cardiovascular and cerebrovascular events (MACCE) in patients with chronic coronary syndrome (CCS) undergoing percutaneous coronary intervention (PCI), using time-varying hazard ratio and time-dependent Receiver Operating Characteristic (ROC) analyses. **Materials and Methods**: A retrospective analysis was conducted on 288 patients diagnosed with CCS from January 2020 to November 2023. The NPS was determined utilizing serum albumin, total cholesterol, the neutrophil-to-lymphocyte ratio (NLR), and the lymphocyte-to-monocyte ratio (LMR). Cox regression, time-varying hazard ratio models, and time-dependent ROC curve analyses were performed to assess both temporal risk patterns and predictive performance. The principal endpoint was the incidence of MACCE. **Results**: Major adverse cardiovascular and cerebrovascular events (MACCE) occurred in 69 individuals, representing 23.4% of the total cohort. Both high NPS and elevated uric acid were independently associated with an increased risk of MACCE. The integration of the NPS with uric acid showed superior discriminative and reclassification capabilities compared to the use of each marker independently (*p* < 0.05 for all). Time-varying hazard ratio analyses demonstrated that the prognostic impact of the NPS was more pronounced in the early follow-up, while the effect of uric acid became stronger in the late phase. Time-dependent ROC analyses confirmed that the combined use of the NPS and uric acid provided superior predictive accuracy compared with either parameter alone across the follow-up period. **Conclusions**: NPS and uric acid offer complementary prognostic information in CCS. Their combined assessment improves long-term risk stratification, while time-varying and time-dependent analyses reveal that their predictive effects evolve dynamically throughout follow-up. This integrated evaluation may improve clinical decision-making and risk stratification in routine practice.

## 1. Introduction

Chronic coronary syndrome (CCS) signifies a stable stage of coronary artery disease (CAD), during which patients are at persistent risk for recurrent ischemic incidents and cardiovascular consequences [1]. Although conventional risk factors and scoring systems have been employed for prognostication, innovative indicators that indicate systemic inflammation, metabolic health, and oxidative stress may provide further insight [2].

The Naples Prognostic Score (NPS), which includes serum albumin, cholesterol, neutrophil-to-lymphocyte ratio (NLR), and lymphocyte-to-monocyte ratio (LMR), has demonstrated potential in forecasting outcomes in several cardiovascular and oncological contexts [3,4,5,6,7]. Concurrently, elevated serum uric acid levels have been independently associated with heightened cardiovascular morbidity and mortality due to mechanisms including endothelial dysfunction, oxidative stress, and inflammation [7,8,9,10]. The synergistic prognostic value of NPS and uric acid in patients with CCS is still inadequately investigated. This study sought to assess if the incorporation of NPS and serum uric acid levels can improve the prediction of long-term major adverse cardiovascular and cerebrovascular events (MACCE) in patients with CCS undergoing percutaneous coronary intervention (PCI).

## 2. Materials and Methods

### 2.1. Patient Population, Study Design, and Data Acquisition

This retrospective cohort analysis encompassed 288 patients diagnosed with coronary artery disease who received PCI between January 2020 and November 2023 at the Department of Cardiology, School of Medicine, Balikesir University. Clinical, biochemical, and demographic information was obtained from hospital records. The serum concentrations of neutrophils, lymphocytes, platelets, hemoglobin, CRP, uric acid, and albumin, together with lipid profiles and markers of liver and kidney function, were evaluated utilizing a Beckman UniCel DxC 800 Synchron autoanalyzer (Beckman Coulter Inc., CA, USA) and suitable tubes. The NPS was determined using established thresholds: albumin < 4 g/dL (1 point), total cholesterol < 180 mg/dL (1 point), NLR ≥ 2.96 (1 point), and LMR < 4.44 (1 point). The overall NPS varied between 0 and 4 [11].

### 2.2. Criteria for Inclusion and Exclusion

Patients with left main disease, current infections, malignancies, autoimmune disorders, systemic inflammatory conditions such as rheumatoid arthritis, systemic lupus erythematosus, inflammatory bowel disease, and vasculitis, those who are breastfeeding or pregnant, and individuals with incomplete clinical or follow-up records were eliminated. The research adhered to the guidelines of the Declaration of Helsinki and received approval from the Medical Ethics Committee of Balikesir University, School of Medicine (3 July 2025-13/7). The Medical Ethics Committee of Balikesir University, School of Medicine, waived the requirement for informed consent due to the study’s retrospective design.

### 2.3. Details About the Procedure and Pharmacological Treatments

The PCI strategy was determined by the treating physician’s discretion. Patients received 300 mg of aspirin together with either clopidogrel (loading dose of 300 mg) or ticagrelor (loading dose of 180 mg) orally for a minimum of 24 h prior to the surgery, unless they were on long-term aspirin or P2Y12 inhibitors. Following PCI, aspirin at a dosage of 75–100 mg daily was provided indefinitely, while either clopidogrel at 75 mg daily or ticagrelor at 90 mg twice daily was prescribed for a minimum duration of 1 year post-procedure.

### 2.4. Outcomes

The principal objective was the occurrence of long-term MACCE, evaluated at a median follow-up of 27.2 (21.6–38.0) months. MACCE was characterized as a composite measure encompassing all-cause mortality, myocardial reinfarction (inclusive of ST-segment elevation myocardial infarction [STEMI] and non-ST-segment elevation myocardial infarction [NSTEMI]), target vessel revascularization (any subsequent revascularization of the epicardial vessel, comprising both main and side branches), and cerebrovascular incidents (including newly acquired neurological deficits such as stroke or transient ischemic attack, validated by radiological imaging). The composite endpoint was evaluated as the time to the initial event.

Clinical follow-up data were acquired via examination of clinic records or telephonic interviews. Follow-up was performed in person or by telephone every three months during the first year following myocardial infarction and every six months thereafter. Mortality and MACCE outcomes for patients lost to follow-up were verified utilizing data from the National Death Registry and the National Social Security Institution.

### 2.5. Statistical Analysis

Continuous variables were represented as mean ± standard deviation (SD), whilst categorical variables were displayed as counts and percentages. Comparisons of continuous variables between groups were conducted using Student’s *t*-test or the Mann–Whitney U test, depending on the distribution of the data. Categorical variables were analyzed with the chi-squared (χ^2^) test. Univariate and multivariate Cox proportional hazards regression analyses were performed to identify independent determinants of long-term MACCE. Multivariable Cox regression models included variables with a *p*-value < 0.10 in univariate analysis. The proportional hazards (PH) assumption was tested for each covariate using Schoenfeld residuals (cox.zph function in R). R version 4.5.1, (R Foundation for Statistical Computing, Vienna, Austria). Global and variable-specific tests were performed, and scaled Schoenfeld residual plots were visually inspected. Although the proportional hazards assumption for serum uric acid was not statistically violated (*p* = 0.082), we incorporated a time-varying Cox model to account for the expected biological fluctuations in serum uric acid levels over time. This approach allows for a more flexible evaluation of its prognostic significance and reflects the dynamic nature of this biomarker in clinical settings. The proportional hazards assumption was violated for the NPS (*p* = 0.017), indicating that its effect on MACCE risk was not constant over time. The proportional hazards assumption of the Cox model was tested, and given the possibility that the prognostic effects of the NPS and uric acid might vary over time, we additionally performed time-varying hazard ratio analyses. This approach enabled us to evaluate the temporal dynamics of their individual and combined associations with long-term MACCE, providing a more accurate characterization of risk throughout the follow-up period. The cumulative incidence of the major endpoints was assessed with the Kaplan–Meier technique. We assessed the predictive value of the NPS and uric acid independently by computing the areas under the receiver operating characteristic curve (ROC). As conventional ROC analysis does not account for censored survival data or the timing of events, we applied time-dependent ROC curve analyses to evaluate how the discriminative ability of the NPS and uric acid, separately and in combination, changed over the course of follow-up. We employed the DeLong test to compare the area under the curve (AUC) for each of these parameters [12]. Furthermore, the enhanced discriminative capacity of combined NPS and uric acid was assessed utilizing net reclassification improvement (NRI) and integrated discrimination improvement [13]. Subgroup analyses were performed across key clinical variables (age, sex, diabetes, hypertension, renal function, left ventricular function, prior stroke, old myocardial infarction, multivessel disease, and the synergy between percutaneous coronary intervention with Taxus and cardiac surgery [SYNTAX] score) to assess the consistency of associations. Interaction terms were introduced into the Cox models, and *p*-interaction values were reported to evaluate heterogeneity. To evaluate the predictive performance and internal validity of the prognostic model combining the NPS and serum uric acid, we assessed both discrimination and calibration. Discrimination was quantified using the area under the receiver operating characteristic curve (AUC) with 95% confidence intervals. Model optimism and potential overfitting were addressed by performing internal validation with 1000 bootstrap resamples, from which optimism-corrected estimates of AUC were obtained. Additional discrimination indices, including Somers’ Dxy and Nagelkerke’s R^2^, were calculated.

Calibration performance was evaluated by estimating the calibration slope and intercept, along with the Brier score as a global measure of model accuracy. Calibration was further examined by plotting predicted versus observed risks using a bootstrap-corrected calibration curve. Calibration accuracy was summarized using Emax (maximum absolute error) and Eavg (average absolute error) statistics.

A two-tailed *p*-value of less than 0.05 was deemed statistically significant. All statistical analyses were conducted utilizing Stata software version 19 (StataCorp LLC, College Station, TX, USA), SPSS software version 30.0 (IBM Corp., Armonk, NY, USA), and R software (version 4.5.1, R Foundation for Statistical Computing, Vienna, Austria).

## 3. Results

### 3.1. Patient Characteristics

A total of 288 patients were included. Of the participants, 69 experienced MACCE, whereas 219 did not and constituted the control group. The average age of patients in the MACCE group was markedly greater than that of the control group (68.2 ± 9.3 years vs. 58.7 ± 10.5 years, *p* < 0.001). The ratio of male patients was comparable across groups (58% versus 51%, *p* = 0.289, Table 1).

The incidence of hypertension, diabetes mellitus, and prior stroke was markedly elevated in the MACCE group compared to the control group (each *p* < 0.05, Table 1). The MACCE group had a significantly elevated prevalence of old myocardial infarction relative to the control group (28% vs. 5%, *p* < 0.001). Multi-vessel disease occurred more frequently in the MACCE group compared to the control group (32% vs. 19%, *p* = 0.021). LVEF was significantly lower in the MACCE group compared to the control group (42.6% vs. 51.1%, *p* < 0.001). The proportion of patients with a SYNTAX score > 22 was higher in the MACCE group than in the control group (13% vs. 2%, *p* < 0.001, Table 1).

Laboratory results are presented in Table 2. The MACCE group had elevated amounts of white blood cells and neutrophils in comparison to those without MACCE (each *p* < 0.05, Table 2). The NLR was significantly higher in the MACCE group compared to the control group with (3.9 [2.7–5.9] vs. 2.2 [1.7–2.8], *p* < 0.001, Table 2). The MACCE group exhibited a reduced LMR in comparison to the control group [2.3 (1.6–3.2) vs. 3.6 (2.9–4.5), *p* < 0.001, Table 2]. Serum uric acid levels were higher in the MACCE group compared to the control group (6.2 ± 1.3 vs. 5.3 ± 1.2, *p* < 0.001, Table 2), whereas albumin levels were reduced in the MACCE group relative to the control group (3.6 ± 0.3 vs. 3.7 ± 0.2, *p* = 0.023, Table 2). The MACCE group exhibited a superior NPS relative to the non-MACCE group (3 [2,3] vs. 2 [2,3], *p* < 0.001, Table 2).

### 3.2. Prognostic Significance of NPS and Uric Acid Levels in CCS Patients Who Have Undergone PCI

Cox regression analysis revealed multiple significant predictors of MACCE. An elevated NPS was identified as an independent predictor of heightened MACCE risk (hazard ratio [HR]: 1.851, 95% confidence interval [CI]: 1.366–2.506, *p* < 0.001; Table 3). Furthermore, blood uric acid levels were correlated with MACCE in individuals with CCS (HR: 1.275, 95% CI: 1.034–1.571, *p* = 0.023, Table 3).

**Figure 1:**
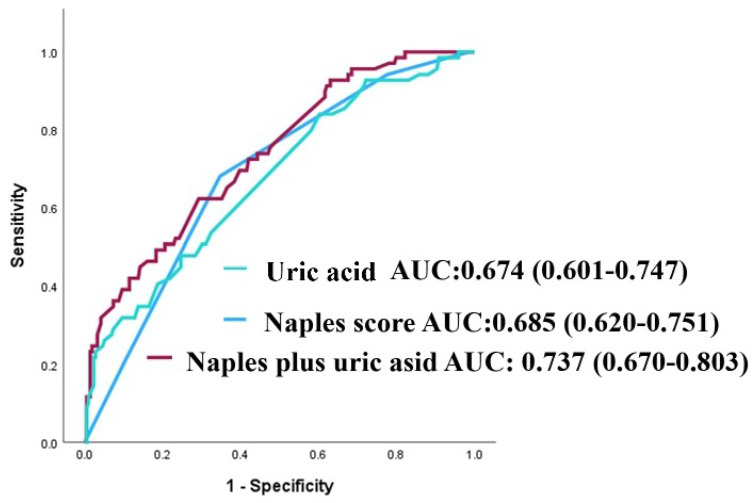
Receiver operating characteristic (ROC) curves for uric acid, the NAPLES score, and their combination in predicting major adverse cardiovascular and cerebrovascular events (MACCE).

The NPS’s discriminative capacity for forecasting MACCE was evaluated by receiver operating characteristic (ROC) analysis. The area under the curve (AUC) for the NPS was 0.685 (95% confidence interval: 0.620–0.751; *p* < 0.001; see Figure 1). The AUC for serum uric acid in predicting MACCE was 0.674 (95% CI: 0.601–0.747; *p* < 0.001; Figure 1). The integration of NPS with uric acid exhibited enhanced predictive efficacy compared to NPS and uric acid individually (AUCs: 0.737 vs. 0.685, z = 1.977, *p* = 0.048; net reclassification improvement (NRI) of 39.0%, *p* < 0.001, and integrated discrimination improvement (IDI) of 0.073, *p* = 0.002; AUCs: 0.737 vs. 0.674, z = 2.244, *p* = 0.025; NRI of 66.5%, *p* < 0.001, and IDI of 0.061, *p* = 0.004), as illustrated in Figure 1.

### 3.3. Survival Analysis

**Figure 2:**
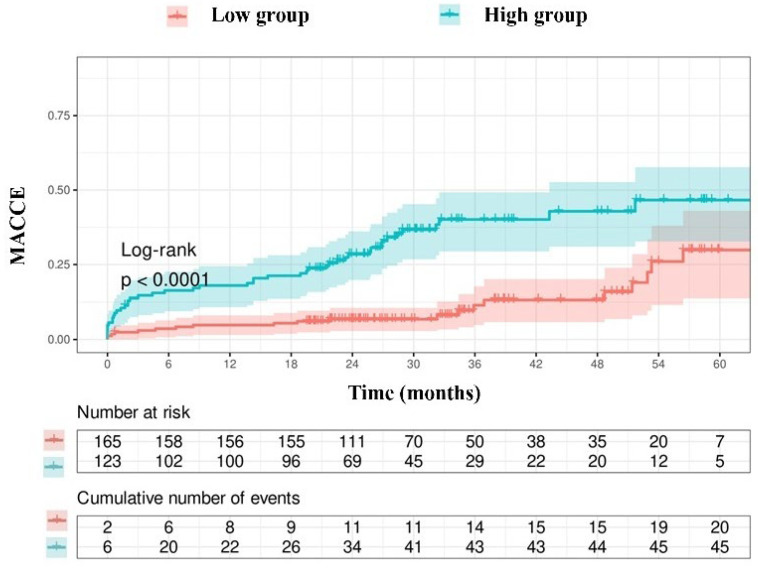
Kaplan–Meier survival curves derived from the NAPLES score.

**Figure 3:**
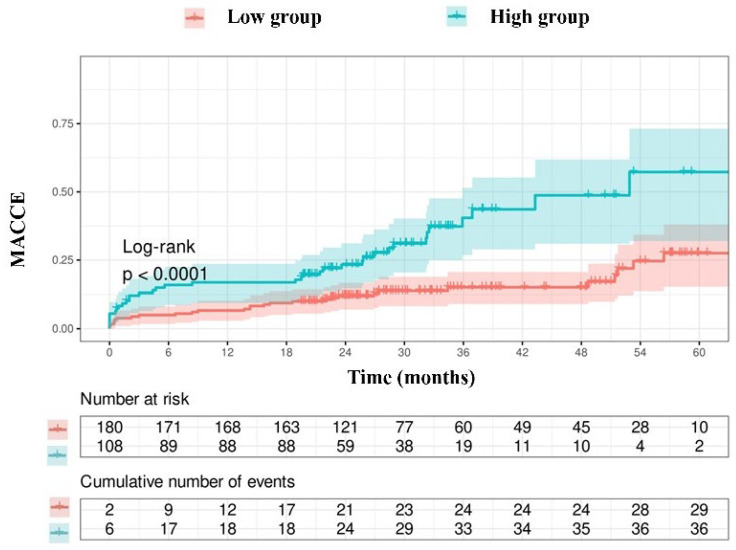
Kaplan–Meier survival curves derived from uric acid concentrations.

**Figure 4:**
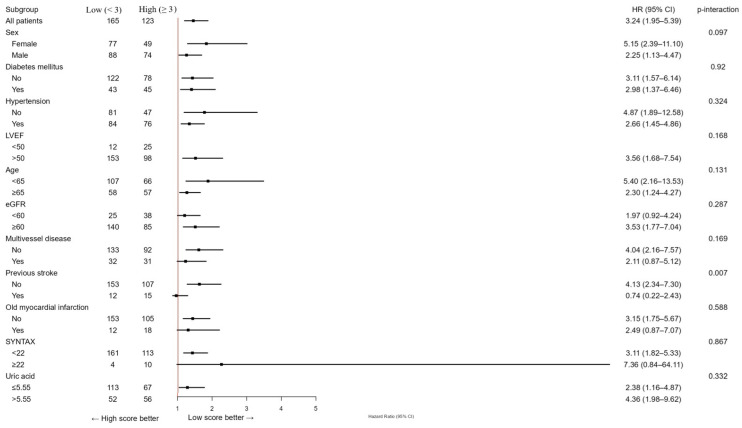
Subgroup analysis of the research cohort utilizing the NAPLES score for the evaluation of MACCE. The model was calibrated for diabetes mellitus, hypertension, previous stroke, historical myocardial infarction, age, left ventricular ejection fraction, multivessel disease, SYNTAX score, glomerular filtration rate, and uric acid levels. HR represents hazards ratio; CI indicates confidence interval, *p* interaction.

**Figure 5:**
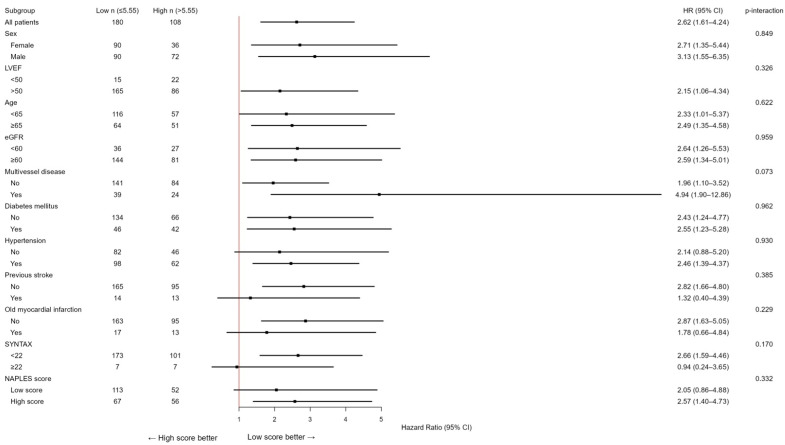
Subgroup analysis of the study population according to uric acid levels in the evaluation of MACCE. The model was calibrated for diabetes mellitus, hypertension, previous stroke, historical myocardial infarction, age, left ventricular ejection fraction, multivessel disease, SYNTAX score, glomerular filtration rate, and NAPLES score. HR represents hazards ratio; CI indicates confidence interval, *p* interaction.

Due to the limited number of patients in the group with an NPS of 0 (*n* = 3), those with an NPS of 0–2 were categorized as the low group (*n* = 165), while those with an NPS of 3–4 were designated as the high group (*n* = 123). The Kaplan–Meier event curves for MACCE categorized by NPS groups are illustrated in Figure 2. The elevated group had a greater MACCE rate than the diminished group (38% vs. 13%, *p* < 0.001, Figure 2). The threshold for uric acid was established at 5.55, exhibiting 54% sensitivity and 68% specificity. Survival curves utilizing this cut-off point indicated that the high group was correlated with elevated MACCE (Figure 3). The subgroup analysis of the study population based on the NPS and uric acid levels is illustrated in Figure 4 and Figure 5.

In subgroup analyses, the prognostic effect of the NPS was generally consistent across major clinical strata (sex, diabetes, hypertension, renal function, left ventricular function, multivessel disease, and age), with no significant interactions (all *p* for interaction >0.05), except for prior stroke (*p* = 0.007). Among patients without stroke, the NPS strongly predicted adverse outcomes (HR 4.13, 95% CI 2.34–7.30), whereas its prognostic impact was attenuated in those with prior cerebrovascular events (HR 0.74, 95% CI 0.22–2.43). Similarly, the association between elevated serum uric acid (>5.55 mg/dL) and adverse outcomes was consistent across all subgroups, with no significant interactions (all *p* > 0.05). A trend toward a stronger effect was observed in patients with multivessel disease, although the interaction was not statistically significant (*p* = 0.073).

### 3.4. Time-Varying HRs and Time-Dependent AUCs

**Figure 6:**
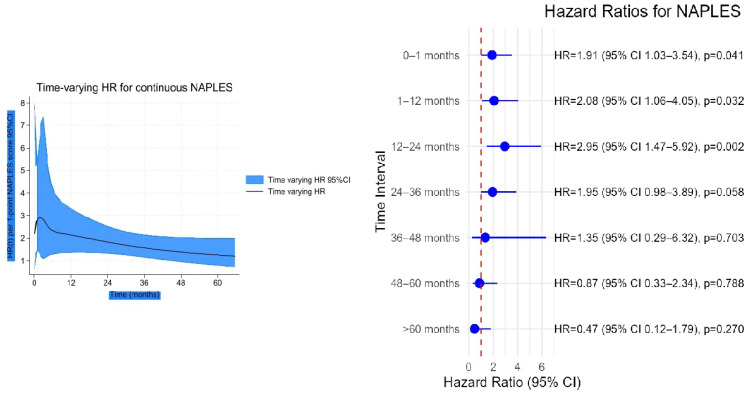
Time-varying hazard ratio (HR) for continuous NAPLES score with 95% confidence intervals.

**Figure 7:**
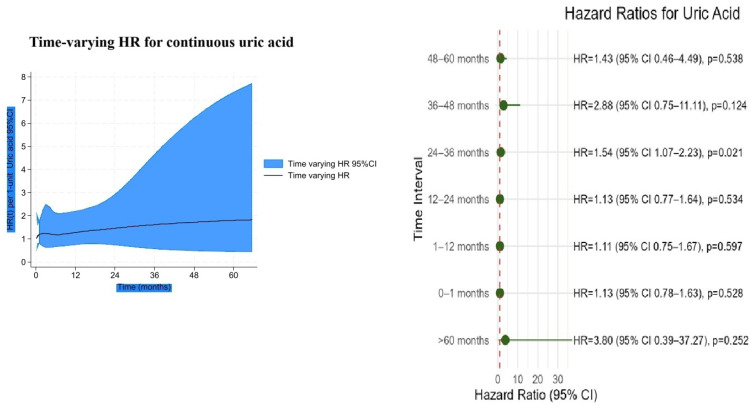
Time-varying hazard ratio for continuous uric acid with 95% confidence intervals.

In the time-varying Cox analysis, the prognostic effect of the NPS was strongest during the early follow-up, with hazard ratios (HRs) exceeding 2 in the first year, and gradually declined thereafter, approaching unity by 60 months (Figure 6). In contrast, uric acid showed minimal risk association at baseline, but its effect increased steadily over time, reaching an HR of nearly 2 at 5 years (Figure 7). Confidence intervals widened markedly after 36 months, indicating reduced precision due to the decreasing number of patients at risk.

**Figure 8:**
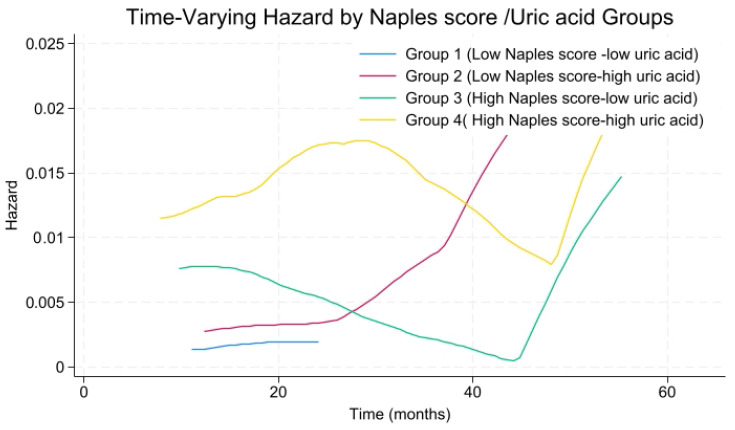
Time-varying hazard rate for NAPLES score-uric acid risk groups.

Time-varying hazard analysis revealed that patients with high NPS and high uric acid levels exhibited a rapid increase in MACCE risk during the early follow-up period, reaching a peak at approximately 20–30 months, after which the risk slightly declined and stabilized (Figure 8). Patients with high NPS but low uric acid demonstrated intermediate-to-high risk in the early period, lower than the high/high group but still elevated, gradually decreasing over time. The low NPS but high uric acid group had moderate early risk, slightly higher than the reference group, with a slow increase followed by stabilization. Finally, the low NPS and low uric acid group remained at consistently low risk throughout follow-up.

**Figure 9:**
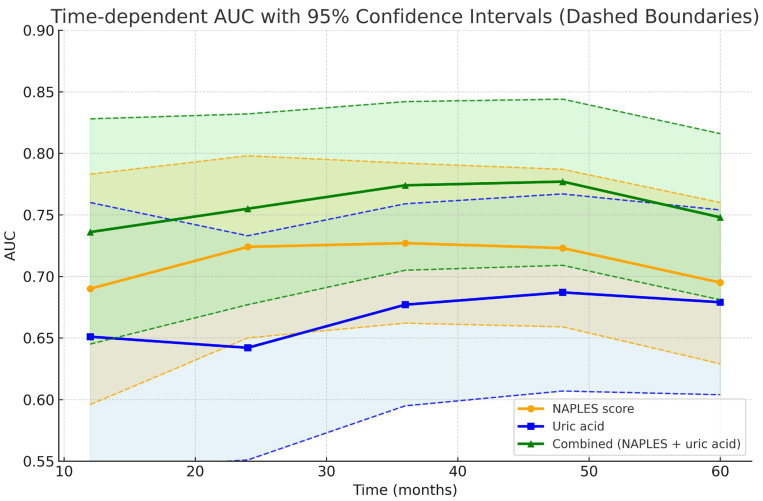
Time-dependent AUC curves with 95% confidence interval.

**Figure 10:**
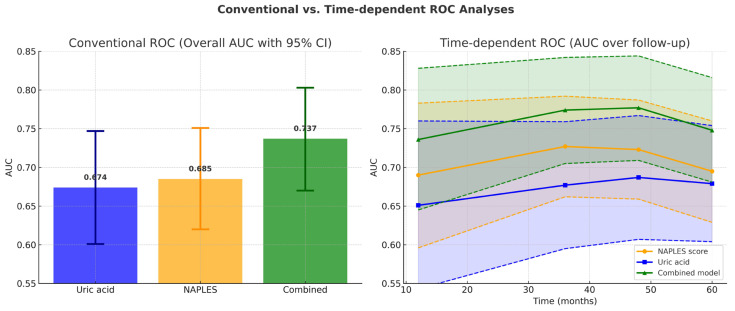
Conventional versus time-dependent ROC analyses. (**Left panel**): Bar graph showing overall AUC values from conventional ROC analysis. The combined model (AUC = 0.737, 95% CI 0.670–0.803) demonstrated superior discrimination compared with the NAPLES score (AUC = 0.685, 95% CI 0.620–0.751) and uric acid alone (AUC = 0.674, 95% CI 0.601–0.747). Error bars represent 95% confidence intervals. (**Right panel**): Time-dependent ROC analysis across 12, 24, 36, 48, and 60 months. The combined model consistently showed numerically higher AUC values over time, achieving significant improvement over uric acid from 36 months onward and over the NAPLES score at 60 months. Shaded areas denote 95% confidence intervals, and dashed lines indicate CI boundaries.

As illustrated in Figure 1 in conventional ROC analysis, the combined model demonstrated superior discrimination compared with the NPS. In time-dependent analyses, however, this advantage was modest in the early and mid-term follow-up and reached statistical significance only at 60 months, suggesting that the incremental prognostic value of uric acid emerges predominantly in the late phase (Figure 9 and Figure 10).

### 3.5. Model Performance and Internal Validation

**Figure 11:**
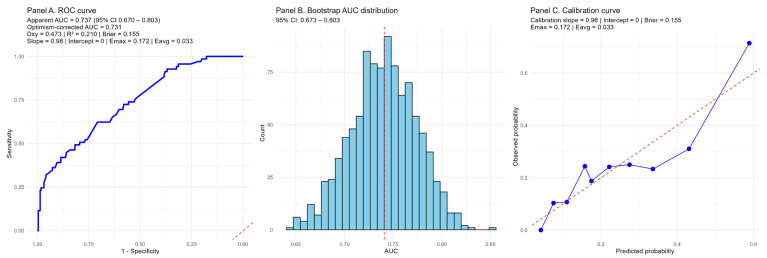
Internal validation of the prognostic model combining the NAPLES score and serum uric acid. (**Panel A**) shows the receiver operating characteristic (ROC) curve with an apparent AUC of 0.737 (95% CI 0.670–0.803) and an optimism-corrected AUC of 0.731 after 1000 bootstrap resamples. Discrimination indices (Dxy = 0.473, R^2^ = 0.210) and the Brier score (0.155) indicate acceptable model performance. (**Panel B**) depicts the distribution of AUC values from bootstrap resampling, confirming stability of discrimination with minimal overfitting. (**Panel C**) presents the calibration curve, demonstrating good agreement between predicted and observed risks (calibration slope ≈ 0.98, intercept ≈ 0, Emax = 0.172, Eavg = 0.033). Blue line is calibration curve of the model, red line is ideal calibration curve.

The prognostic model combining the NPS and serum uric acid demonstrated acceptable discriminatory ability for predicting long-term MACCE. The apparent AUC was 0.737 (95% CI: 0.673–0.803), while internal validation with 1000 bootstrap resamples yielded an optimism-corrected AUC of 0.731, suggesting minimal overfitting and stable model performance. Additional indices (Dxy = 0.473, R^2^ = 0.210, Brier score = 0.155, calibration slope ≈ 0.98, intercept ≈ 0, Emax = 0.172, Eavg = 0.033) further supported the robustness of the model. Calibration analysis showed good agreement between predicted and observed probabilities, as shown by the calibration curve (Figure 11).

## 4. Discussion

These findings suggest that the prognostic significance of inflammatory-nutritional status (as reflected by the NPS) and metabolic burden (uric acid) may differ across the clinical trajectory. The NPS appears to capture early risk, potentially reflecting patients’ baseline vulnerability, whereas uric acid demonstrates a cumulative, long-term deleterious effect. Importantly, the combined assessment of both parameters provided superior risk stratification, with patients harboring high values of both indices experiencing the greatest long-term hazard. This synergistic effect underscores the value of integrating metabolic and inflammatory-nutritional markers into prognostic models for patients undergoing longitudinal follow-up.

From a statistical perspective, the different patterns observed in conventional and time-dependent ROC analyses provide further insights into the prognostic dynamics of the studied markers. Conventional ROC analysis, which integrates all events during follow-up, demonstrated a global superiority of the combined model over both NPS and uric acid. However, time-dependent ROC analyses revealed that this incremental value over the NPS score became statistically significant only at 60 months, whereas superiority over uric acid was evident from 36 months onward. These findings suggest that the NPS predominantly captures early and mid-term risk, while uric acid contributes more strongly to long-term prognostic discrimination.

The NPS incorporates indicators of systemic inflammation (NLR, LMR) and nutritional status (albumin, cholesterol), both of which are increasingly acknowledged as significant factors in cardiovascular risk. Inflammatory processes are pivotal in the start and evolution of atherosclerotic plaques, with indicators like NLR linked to unfavorable outcomes in various cardiovascular contexts. Hypoalbuminemia and hypocholesterolemia are markers of malnutrition and systemic illness, both linked to poorer prognoses in cardiovascular patients. A multicenter retrospective investigation of 1887 patients with ST-segment elevation myocardial infarction (STEMI) revealed that a high NPS (3–4) correlated with a significant increase in in-hospital adverse events and all-cause death during follow-up [14]. A comprehensive review and meta-analysis of STEMI patients (*n* ≈ 12,785) corroborated elevated risks of mortality, shock, acute renal damage, and no-reflow in individuals with high levels [15]. A recent study of STEMI patients indicated that NPS was independently associated with in-hospital mortality [16].

Despite the limited data on CCS, these STEMI findings corroborate the NPS’s capacity to assess inflammation, malnutrition, and worse prognosis in ischemic heart disease. The elements of the score—hypoalbuminemia, dyslipidemia, and elevated inflammatory ratios—are established risk factors for cardiovascular events, so NPS is affirmed as a valuable integrative biomarker.

Conversely, uric acid serves as both a marker and a mediator of oxidative stress [11]. Hyperuricemia has been associated with endothelial dysfunction, heightened oxidative stress, and inflammation, which are fundamental to the pathogenesis of coronary artery disease (CAD) [14]. The pro-inflammatory properties of uric acid may aggravate vascular injury and enhance plaque instability, hence facilitating the incidence of MACCE. Numerous studies indicate that increased uric acid levels independently forecast negative cardiovascular outcomes, even when accounting for conventional risk variables [17,18]. A meta-analysis of 41 studies indicated that elevated uric acid levels in individuals with acute myocardial infarction correlate with a heightened risk of both in-hospital and long-term severe adverse cardiovascular events [19]. Hyperuricemia has been demonstrated to correlate with an elevated risk of significant cardiovascular events in individuals with chronic coronary syndrome undergoing percutaneous coronary intervention [20].

The biological components of the NPS and serum uric acid appear to exert synergistic effects, reinforcing one another’s prognostic relevance. This interplay may contribute to improved risk prediction by capturing complementary aspects of metabolic, nutritional, and inflammatory pathways. The NPS reflects the interaction between dietary status and systemic inflammation, both of which are closely linked to adverse cardiac remodeling, oxidative stress, and impaired vascular repair [21,22]. In parallel, elevated uric acid levels are associated with endothelial dysfunction, activation of the renin–angiotensin system, and promotion of a pro-inflammatory milieu, thereby reflecting heightened oxidative stress and metabolic demand [23]. Together, these indices highlight distinct yet interconnected biological trajectories that converge to influence cardiovascular risk.

Consistent associations between uric acid and adverse prognosis across clinical subgroups suggest that its risk contribution is largely independent of baseline comorbidities. Although a trend toward a stronger effect was observed among patients with multivessel coronary disease, this interaction did not reach statistical significance, but it raises the possibility of a synergistic relationship between hyperuricemia and advanced atherosclerosis that merits further investigation. Similarly, subgroup analyses demonstrated that the prognostic impact of the NPS was robust across diverse clinical strata, with only limited evidence of effect modification, even among patients with a history of stroke.

Internal validation using bootstrap resampling confirmed that the model combining the NPS and serum uric acid achieved stable discrimination and satisfactory calibration, with minimal overfitting. Taken together, these findings underscore the potential of integrating metabolic and inflammatory–nutritional markers to improve risk stratification, while also emphasizing the need for external validation in independent cohorts before clinical application.

The uniqueness of our study resides in assessing the combined prognostic influence of NPS and uric acid, which, to our knowledge, has not been previously documented in patients with CCS. Our findings indicate that this integrated evaluation yields superior risk classification compared to either indicator individually. This has practical significance, as both NPS and uric acid may be easily acquired through standard blood tests, rendering them accessible instruments for clinical application. From a preventive standpoint, recognizing individuals with elevated NPS and hyperuricemia may assist doctors in implementing more rigorous secondary prevention efforts, encompassing enhanced medicinal therapy, lifestyle alterations, and increased monitoring. Furthermore, novel medicines aimed at uric acid metabolism (such as xanthine oxidase inhibitors) or systemic inflammation may provide additional therapy options for high-risk people.

## 5. Study Limitations

Our research possesses multiple limitations. The retrospective and single-center methodology may restrict the applicability of our findings. We were unable to evaluate the temporal variations in NPS and uric acid, which could offer further prognostic insights. The intentional exclusion of patients with left main coronary artery disease aimed to minimize heterogeneity and prevent confusion arising from the distinct prognostic factors of this category. The outcomes in LMCA disease are mostly determined by anatomical and procedural variables [24,25], perhaps masking the prognostic significance of systemic metabolic or inflammatory markers such as NPS and uric acid. By concentrating on individuals with non-left-main coronary artery disease, our study elucidates the association between these biomarkers and long-term outcomes within a more homogeneous clinical framework. Consequently, our results must be understood within the context of stable or non-left-main coronary syndromes. Although the model demonstrated satisfactory internal performance, its generalizability to other populations and clinical settings remains to be confirmed through external validation in independent cohorts. Moreover, the uric acid cut-off value identified in this study (5.55 mg/dL) was derived using the Youden index, which may reflect sample-specific optimization rather than a universally applicable threshold; thus, established clinical cut-offs (e.g., ≥7.0 mg/dL in men and ≥6.0 mg/dL in women) [8] should also be evaluated in future studies. Since the composite endpoint encompassed all-cause mortality, which is not specific to cardiovascular causes, the associations between the studied predictors and cardiovascular outcomes may have been attenuated. Therefore, the absence of detailed cause-of-death data should be acknowledged as a potential limitation. Also, data regarding uric acid-lowering therapies were unavailable, which may have influenced serum uric acid levels and should be considered when interpreting the results. Furthermore, despite adjusting for multiple variables, residual confounding remains a possibility.

## 6. Conclusions

The integration of the NPS and serum uric acid levels offers a comprehensive and synergistic approach for long-term risk evaluation in CCS patients. Prospective, multicenter investigations are necessary to confirm our findings and investigate the therapeutic implications of addressing inflammation and uric acid in this population.

## Figures and Tables

**Table 1:** Demographic and clinical features of the study population.

Variables	MACCE (−) (*n* = 219)	MACCE (+) (*n* = 69)	*p*-Value
Age	58.7 ± 10.5	68.2 ± 9.3	<0.001
Male, *n* (%)	127 (58)	35 (51)	0.289
Diabetes mellitus, *n* (%)	56 (26)	32 (46)	0.001
Hypertension, *n* (%)	112(51)	48 (70)	0.007
Old myocardial infarction (*n*, %)	11 (5)	19 (28)	<0.001
Previous PCI (*n*, %)	42 (19)	16 (23)	0.469
Previous CABG (*n*, %)	14 (6)	6 (9)	0.518
Previous stroke, *n* (%)	30 (5)	11 (10)	0.021
Hyperlipidemia, *n* (%)	104 (48)	42 (61)	0.053
Family history of coronary artery disease (*n*, %)	37 (17)	13(19)	0.710
Lesions involving arteries			0.264
LAD *n* (%)	118 (54)	39 (857)	
LCx (*n*)	36 (17)	6 (9)	
RCA (*n*)	65 (30)	24 (35)	
Multivessel disease, *n* (%)	41 (19)	22 (32)	0.021
Smoking, *n* (%)	92 (42)	33 (48)	0.395
LVEF (%)	51.1 ± 3.1	42.6 ± 8.5	<0.001
SYNTAX > 22	5 (2)	9 (13)	<0.001
Medical treatment at discharge, *n* (%)			
DAPT, *n* (%)	216 (99)	67 (97)	0.397
Beta blockers, *n* (%)	147 (67)	43 (62)	0.463
ACE/ARB, *n* (%)	85 (39)	34 (49)	0.124
Statin *n* (%)	216 (99)	66 (96)	0.131
MACCE *n* (%)			
All-cause mortality *n* (%)	-	9 (3)	
Target vessel revascularization *n* (%)	-	25 (9)	
Myocardial reinfarction *n* (%)	-	8 (3)	
Repeated stroke *n* (%)	-	3 (1)	
Hospitalization with heart failure *n* (%)	-	24 (8)	

**Abbreviations:** LVEF: left ventricular ejection fraction, ACE/ARB: angiotensin-converting enzyme inhibitor/angiotensin receptor blockers, PCI, Percutaneous coronary intervention; CABG, coronary artery bypass grafting; DAPT, dual antiplatelet treatment; LM, left main; LAD, left anterior descending; LCx, left circumflex; RCA, right coronary artery; SYNTAX, the synergy between percutaneous coronary intervention with Taxus and cardiac surgery.

**Table 2:** Laboratory and Index Parameters of the Study Groups.

Variables	MACCE (−) (*n* = 219)	MACCE (+) (*n* = 69)	*p*-Value
Haemoglobin (g/dL)	13.1 ± 1.8	13.1 ± 1.8	0.858
Platelets (10^3^/µL)	255.7 ± 78.3	244.1 ± 72.2	0.234
Neutrophil (10^3^/µL)	5.2 ± 2.0	6.6 ± 3.0	<0.001
Lymphocyte (10^3^/µL)	2.3 ± 1.1	1.6 ± 0.6	<0.001
Monocyte (10^3^/µL)	0.6 (0.5–0.8)	0.6 (0.5–0.9)	0.094
White blood cell (10^3^/µL)	8.5 ± 2.6	9.3 ± 3.3	0.037
NLR	2.2 (1.7–2.8)	3.9 (2.7–5.9)	<0.001
LMR	3.6 (2.9–4.5)	2.3 (1.6–3.2)	<0.001
Glucose (mg/dL)	118.7 ± 45.5	129.6 ± 60.3	0.112
Total Cholesterol (mg/dL)	190.1 ± 46.0	196.9 ± 49.7	0.294
LDL-C (mg/dL)	110.1 ± 38.2	116.5 ± 35.0	0.219
HDL-C (mg/dL)	48.1 ± 12.8	47.8 ± 9.7	0.817
Triglyceride (mg/dL)	131 (100–183)	143 (105–236)	0.053
GFR (mL/min/1.73 m^2^)	80.1 ± 19.9	62.8 ± 24.4	<0.001
Albumin (g/dL)	3.7 ± 0.2	3.6 ± 0.3	0.023
Uric acid (mg/dL)	5.3 ± 1.2	6.2 ± 1.3	<0.001
NAPLES score	2 (2–3)	3 (2–3)	<0.001

**Abbreviations:** GFR: Glomerular Filtration Rate, NLR: neutrophil to lymphocyte ratio, LMR: lymphocyte to monocyte ratio, HDL-C: high-density lipoprotein cholesterol, LDL-C: low-density lipoprotein cholesterol.

**Table 3:** Predictors of mortality in univariate and multivariate analysis.

	Univariate			Multivariate		
Variables	HR	95%CI	*p*	HR	95%CI	*p*
Age	1.071	1.045–1.098	<0.001	1.036	1.008–1.064	0.011
Diabetes mellitus	2.082	1.296–3.343	0.002			
Hypertension	1.967	1.174–3.297	0.010			
Old myocardial infarction	1.453	0.948–2.226	0.086			
Previous stroke	1.023	1.010–10.37	<0.001			
Multivessel disease	1.762	1.059–2.931	0.029			
Hyperlipidemia	1.451	0.894–2.355	0.132			
LVEF	0.903	0.883–0.924	<0.001	0.911	0.874–0.948	<0.001
Syntax score > 22	3.745	1.845–7.602	<0.001			
Triglyceride	1.002	0.999–1.004	0.127			
GFR	0.970	0.960–0.980	<0.001	0.987	0.977–0.997	0.011
Neutrophil count	1.184	1.091–1.284	<0.001			
Lymphocyte count	0.388	0.271–0.555	<0.001			
Monocyte count	1.181	0.882–1.583	0.264			
NLR	1.137	1.083–1.193	<0.001			
LMR	0.703	0.595–0.830	<0.001			
Albumin	0.927	0.850–1.011	0.085			
Uric acid	1.524	1.309–1.774	<0.001	1.252	1.018–1.539	0.033
Naples score	1.765	1.368–2.276	<0.001	1.792	1.328–2.418	<0.001

**Abbreviations:** HR: Hazard Ratio; CI: Confidence Interval; GFR: Glomerular Filtration Rate, NLR: neutrophil to lymphocyte ratio, LMR: lymphocyte to monocyte ratio; SYNTAX, the synergy between percutaneous coronary intervention with Taxus and cardiac surgery; LVEF: left ventricular ejection fraction.

## Data Availability

The data supporting this study’s findings are available from the corresponding author upon reasonable request.
